# Impact of Sleep Duration on Depression and Anxiety After Acute Ischemic Stroke

**DOI:** 10.3389/fneur.2021.630638

**Published:** 2021-03-26

**Authors:** Fei Liu, Yang Yang, Shuo Wang, Xiao-Li Zhang, An-Xin Wang, Xiao-Ling Liao, Hong-Juan Fang, Yue Qu, Wei-Guo Ma, Ning Zhang, Chun-Xue Wang, Yong-Jun Wang

**Affiliations:** ^1^Department of Neurology, Beijing Tiantan Hospital, Capital Medical University, Beijing, China; ^2^China National Clinical Research Center for Neurological Diseases, Beijing Tiantan Hospital, Capital Medical University, Beijing, China; ^3^Department of Neuropsychiatry and Behavioral Neurology and Clinical Psychology, Beijing Tiantan Hospital, Capital Medical University, Beijing, China; ^4^Department of Endocrinology, Beijing Tiantan Hospital, Capital Medical University, Beijing, China; ^5^Independent Researcher, Ithaca, NY, United States; ^6^Beijing Anzhen Hospital, Capital Medical University, Beijing, China; ^7^Collaborative Innovation Center for Brain Disorders, Beijing Institute of Brain Disorders, Capital Medical University, Beijing, China

**Keywords:** sleep duration, post-stroke anxiety, post-stroke depression, ischemic stroke, prognosis

## Abstract

**Background:** Abnormal sleep duration predicts depression and anxiety. We seek to evaluate the impact of sleep duration before stroke on the occurrence of depression and anxiety at 3 months after acute ischemic stroke (AIS).

**Methods:** Nationally representative samples from the Third China National Stroke Registry were used to examine cognition and sleep impairment after AIS (CNSR-III-ICONS). Based on baseline sleep duration before onset of stroke as measured by using the Pittsburgh Sleep Quality Index (PSQI), 1,446 patients were divided into four groups: >7, 6–7, 5–6, and <5 h of sleep. Patients were followed up with the General Anxiety Disorder-7 (GAD-7) and Patient Health Questionnaire-9 (PHQ-9) for 3 months. Poststroke anxiety (PSA) was defined as GAD-7 of ≥5 and poststroke depression (PSD) as PHQ-9 of ≥5. The association of sleep duration with PSA and PSD was evaluated using multivariable logistic regression.

**Results:** The incidences of PSA and PSD were 11.2 and 17.6% at 3 months, respectively. Compared to a sleep duration of >7 h, 5–6 h, and <5 h of sleep were identified as risk factors of PSA [odds ratio (OR), 1.95; 95% confidence interval (CI), 1.24–3.07; *P* < 0.01 and OR, 3.41; 95% CI, 1.94–6.04; *P* < 0.01) and PSD (OR, 1.47; 95% CI, 1.00–2.17; *P* = 0.04 and OR, 3.05; 95% CI, 1.85–5.02; *P* < 0.01), while 6–7 h of sleep was associated with neither PSA (OR, 1.09; 95% CI, 0.71–1.67; *P* = 0.68) nor PSD (OR, 0.92; 95% CI, 0.64–1.30; *P* = 0.64). In interaction analysis, the impact of sleep duration on PSA and PSD was not affected by gender (*P* = 0.68 and *P* = 0.29, respectively).

**Conclusions:** Sleep duration of shorter than 6 h was predictive of anxiety and depression after ischemic stroke.

## Introduction

Post-stroke depression (PSD) and post-stroke anxiety (PSA) are among the most common complications of acute ischemic stroke (AIS), seen in 31 and 35% of stroke survivors, respectively (1). Individuals with PSD and PSA have poor prognosis with higher risk of mortality (2–4). Due to the lack of effective treatment for PSA or PSD, identification of the predictors for PSA or PSD may lead to discovery of modifiable risk factors, which is of great clinical relevance.

Previous studies have found that short duration of sleep was associated with chronic diseases. Sleep duration of <7 h has been shown to be closely related to the development of stroke, diabetes mellitus, and depression (5). Old and middle-aged people with <5 h (1.69 times) and 5–6 h (1.48 times) of sleep had a higher risk of depression compared to those sleeping for 7–8 h, and those who sleep for >9 h had no significant risk (6).

Up to date, the impact of sleep duration prior to stroke on the occurrence of anxiety and depression after ischemic stroke is still elusive and undefined. Moreover, the duration of sleep can be a potential therapeutic target, despite that most risk factors for PSA or PSD are not modifiable, such as gender, stroke severity, frontal pole lesion, communication disorders, cognitive impairment, chronic pain, and lack of social support (4, 7–10). Therefore, this study aims to investigate the impact of the duration of sleep before stroke on the occurrence of anxiety and depression at 3 months after ischemic stroke.

## Methods

### Study Settings and Participants

The Third China National Stroke Registry (CNSR-III) is a national prospective registry on the etiology, imaging, and biomarkers of ischemic stroke and transient ischemic attack (TIA) (http://paper.ncrcnd.ttctrc.com/default/project-detail?id=253). In CNSR-III, the data of 15,166 stroke patients were collected from a total of 201 study sites from August 2015 to March 2018 (11). The present study uses the data of AIS patients derived from the Impairment of Cognition and Sleep after AIS or Transient Ischemic Attack Study in Chinese Patients (ICONS), which is a subgroup study of CNRS-III on cognition and sleep (12). AIS was diagnosed according to the World Health Organization (WHO) diagnostic criteria and confirmed with craniocerebral magnetic resonance imaging (MRI) or computed tomography (CT) during hospitalization.

Patients were included in this study if they fulfilled all the following criteria: (1) 18 years of age or older, (2) within 7 days from the onset of ischemic stroke symptoms to enrollment, and (3) completed the baseline and follow-up tests.

Individuals were excluded if they met one of the following criteria: (1) silent cerebral infarction without symptoms or signs; (2) illiterate; (3) prior history of stroke, sleep disorder, depression, anxiety, schizophrenia, or cognitive impairment (as diagnosed by physicians); or (4) could not cooperate because of hearing loss, vision dysfunction, aphasia [National Institutes of Health Stroke Scale (NIHSS) item 9 > 2], and consciousness disturbance (NIHSS item 1a > 1 or 1b > 1).

The study protocol was approved by the ethics committees of participating hospitals, and written consent was obtained from all participants.

### Data Collection and Data Management

Sociodemographic information, medical history, and scale measurements were collected from medical records and clinical interviews by trained neurologists.

Social demographic variables include age, gender, marital status (married vs. other), education (below high school vs. high school and above), employment status (employed, retired, unemployed, or unknown), and monthly income (high level, >2,300 RMB vs. low level, ≤ 2,300 RMB).

Lifestyle variables include body mass index (BMI), physical activity conditions including heavy labor, housework, transportation, and leisure exercise (yes or no), current smoking (Do you smoke habitually?) (yes or no), and alcohol use (Do you drink habitually?) (yes or no).

Past medical history include hypertension, hyperlipidemia, diabetes, heart disease, migraine, and family history of stroke (yes vs. no).

The modified Rankin Scale (mRS) was used to measure the functional status. The National Institutes of Health Stroke Scale (NIHSS) was used to assess severity of stroke, including motor disorders (NIHSS item 5a or 5b or 6a or 6b ≥1), sensory disorders (NIHSS item 8 ≥ 1), and language disorders (NIHSS item 9 or 10 ≥ 1). The status of anxiety and depression at baseline and the types of wards/specialties during hospitalization [stroke unit, intensive care unit (ICU) and other wards] were also collected.

The duration of sleep prior to the onset of stroke was assessed during the hospitalization using the question “During the past month, how many hours of actual sleep did you get at night?” in the Pittsburgh Sleep Quality Index (PSQI). In conformance to the scoring principle as described in Component 3 of PSQI, the duration of sleep was categorized into four groups: >7, 6–7, 5–6, and <5 h (13).

Data on PSA and PSD were collected by face-to-face interview at 3 months after stroke. General Anxiety Disorder-7 (GAD-7) was used to assess the severity of anxiety during the past 2 weeks, with a total score of 21 points (whereby 0–4 denotes no anxiety and 5–9, 10–14, and 15–21 represent mild, moderate, and severe anxiety) (14). PSA was defined as a GAD-7 score of ≥5.

Patient Health Questionnaire-9 (PHQ-9) was used to evaluate depression during the past 2 weeks, with a total score of 27 points (in which 0–4 represents no depression, while 5–9, 10–14, 15–19, and 20–27 are taken as mild, moderate, moderately severe, and severe depression) (15). PSD was defined as a PHQ-9 score of ≥5.

### Statistical Analysis

Continuous variables were expressed as mean ± standard deviation or median (interquartile range, IQR), and analyzed using the Kruskal–Wallis test or Wilcoxon rank-sum test. Categorical variables were presented as numbers (percentages) and analyzed using chi-square test or Fisher's exact probability test.

Multivariate logistic regression was performed to evaluate the impact of the duration of sleep prior to onset of stroke on the occurrence of PSA or PSD, in which sleep duration of >7 h was taken as the reference group. The results were presented with odds ratios (ORs) and their 95% confidence intervals (95% CI). Three models were built to adjust potential covariates (e.g., age, level of education, employment status, smoking, heart disease), which may affect PSD/PSA according to previous studies (16–19). In Model 1, the ORs were adjusted for age and gender. Model 2 were adjusted for social demographic, lifestyle, and medical history variables beside gender and age. In Model 3, functional scales and the ward types or specialties during hospitalization were adjusted in addition to Model 2 adjustments. In addition, propensity score matching was performed to balance the four groups of sleep duration on other variables.

To test the linear trend, we entered zero, one, two and three of each sleep duration as a continuous variable in the model.

Potential interaction was further examined with stratification by gender.

Data were analyzed using SAS V9.4 (SAS Institute, Cary, NC). All tests were two-sided, and a *P* < 0.05 was considered statistically significant.

## Results

### Baseline Patient Characteristics

**Figure 1:**
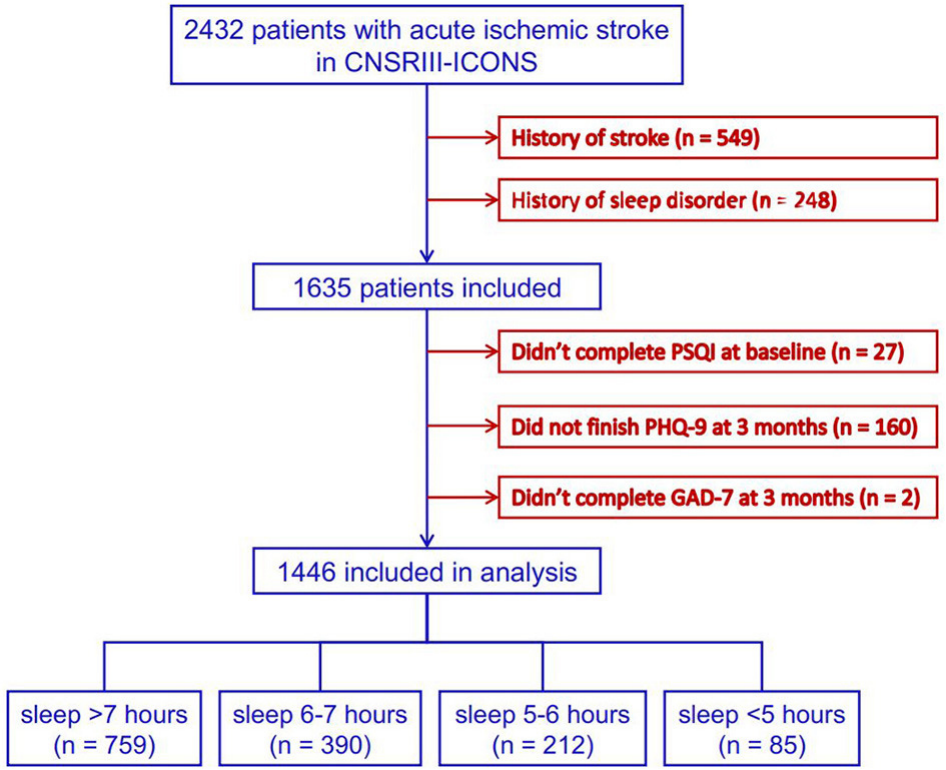
Flowchart of patient enrollment. CNSR-III-ICONS, China National Stroke Registry-III-the impairment of cognition and sleep after acute ischemic stroke or transient ischemic attack in Chinese patient study; PSQI, Pittsburgh Sleep Quality Index; PHQ-9, Patient Health Questionnaire-9; GAD-7, General Anxiety Disorder-7.

Data of 2,432 patients with AIS were collected in the CNSR-III-ICONS from 40 participating sites. After excluding 549 patients with stroke history, 248 with sleep disorder history, 27 without complete baseline information, and 162 who failed to complete the follow-up at 3 months, a total of 1,446 patients were included in this analysis (Figure 1).

The numbers of patients in the four groups of sleep duration after ischemic stroke were as follows: >7 h, 759 (52.5%); 6–7 h, 390 (27.0%); 5–6 h, 212 (14.7%); and <5 h, 85 (5.9%), respectively.

The four groups differed significantly in age, education level, history of hypertension and migraine, family history of stroke, ward type/specialty, and the status of depression and anxiety at baseline. Patients in the 5–6 h of sleep group were aged 61.9 ± 11.3 years on average and significantly older than the group who slept for >6 h (*P* < 0.01). Higher educational level was more common in the groups with 6–7 and <5 h of sleep compared to the group with >7 h of sleep (*P* < 0.01 and *P* = 0.04). Hypertension and family history of stroke were more common in patients of the <5 h group compared to other groups (all *P* < 0.05). Patients with 5–6 h of sleep were less likely to have motor disorder than patients sleeping for more than 6 h (*P* < 0.01 and *P* = 0.02). More patients admitted in the stroke center and ICU were likely to sleep for 5–6 h rather than >6 h (*P* < 0.01). Patients with a shorter duration of sleep were more likely to have depression and anxiety at baseline (Table 1, Supplementary Table 1).

**Table 1:** Baseline patient characteristics stratified by sleep durations.

Variable^*^	**Duration of sleep**				***P*-value**
	**>7 h** **(*n* = 759, %)**	**6–7 h** **(*n* = 390, %)**	**5–6 h** **(*n* = 212, %)**	**<5 h** **(*n* = 85, %)**	
Male gender	568 (74.8)	275 (70.5)	155 (73.1)	58 (68.2)	0.32
Age, year	59.6 ± 11.1	59.4 ± 10.8	61.9 ± 11.3	61.0 ± 9.7	0.02
Body mass index, kg/m^2^	24.9 ± 3.1	25.2 ± 3.2	25.1 ± 3.2	25.3 ± 3.1	0.72
Married	719 (94.7)	367 (94.1)	206 (97.2)	80 (94.1)	0.40
Education ≥ high school	222 (29.3)	144 (36.9)	73 (34.4)	34 (40.0)	0.02
Employment status					0.13
Employed	472 (62.2)	237 (60.8)	117 (55.2)	49 (57.7)	
Retired	180 (23.7)	88 (22.6)	64 (30.2)	26 (30.6)	
Unemployed	63 (8.3)	43 (11.0)	25 (11.8)	5 (5.9)	
Unknown	44 (5.8)	22 (5.6)	6 (2.8)	5 (5.9)	
High monthly income	260 (34.3)	148 (38.0)	78 (36.8)	31 (36.5)	0.64
Current smoker	297 (39.1)	137 (35.1)	81 (38.2)	30 (35.3)	0.57
Current drinker	154 (20.3)	76 (19.5)	52 (24.5)	16 (18.8)	0.47
Physical activity	523 (68.9)	256 (65.6)	145 (68.4)	59 (69.4)	0.71
Hypertension	427 (56.3)	226 (58.0)	127 (59.9)	62 (72.9)	0.02
Hyperlipidemia	57 (7.5)	30 (7.7)	20 (9.4)	10 (11.8)	0.47
Diabetes	143 (18.8)	92 (23.6)	51 (24.1)	21 (24.7)	0.13
Heart disease	81 (10.7)	45 (11.5)	30 (14.2)	16 (18.8)	0.10
Migraine	7 (0.9)	11 (2.8)	3 (1.4)	5 (5.9)	<0.01
Family history of stroke	125 (16.5)	77 (19.7)	26 (12.3)	26 (30.6)	<0.01
mRS at baseline	1.7 ± 1.2	1.9 ± 1.3	1.7 ± 1.3	1.6 ± 1.2	0.08
NIHSS at baseline	3.4 ± 3.0	3.5 ± 2.9	3.6 ± 3.6	3.3 ± 2.9	0.82
Motor disorders	443 (58.4)	219 (56.2)	99 (46.7)	43 (50.6)	0.01
Sensory disorders	189 (24.9)	100 (25.6)	52 (24.5)	29 (34.1)	0.31
Language disorders	331 (43.6)	170 (43.6)	100 (47.2)	38 (44.7)	0.81
Ward types/specialties					<0.01
Stroke unit	114 (15.0)	62 (15.9)	53 (25.0)	19 (22.4)	
ICU	11 (1.5)	6 (1.5)	7 (3.3)	1 (1.2)	
Other	634 (83.5)	322 (82.6)	152 (71.7)	65 (76.5)	
Depression at baseline	2.3 ± 3.2	2.3 ± 3.3	3.4 ± 4.0	6.3 ± 5.2	<0.01
Anxiety at baseline	1.8 ± 3.0	1.8 ± 2.9	2.5 ± 3.9	4.0 ± 5.4	<0.01

* *All values are expressed as number (percentage) except age, body mass index, mRS, NIHSS at baseline, depression, and anxiety at baseline*.

*mRS, Modified Ranking Scale; NIHSS, National Institutes of Health Stroke Scale; ICU, Intensive Care Unit; Other, wards/specialties exclusive of the stroke unit and ICU*.

### Impact of Sleep Duration on PSA at 3 Months

At 3 months after acute ischemic stroke, 162 patients (11.2%) had a GAD-7 score of ≥5. With all potential confounding factors adjusted in Model 3, there was no significant difference between the groups with 6–7 and >7 h of sleep. However, patients who slept for 5–6 h and <5 h were more likely to develop PSA compared to the reference group (OR, 1.95; 95% CI, 1.24–3.07, *P* < 0.01 and OR, 3.41; 95% CI, 1.94–6.04; *P* < 0.01; respectively). A significant inverse correlation was observed between the prevalence of PSA and sleep duration (*P* for trend <0.01) (Table 2). The same trend persisted after adjustment with propensity score matching (Supplementary Table 2).

**Table 2:** Impact of sleep duration on anxiety at 3 months after stroke.

**Sleep duration (h)**	**Odds ratio (95% confidence interval**, ***P*****-value)**		
**Total (*n* = 1,446)**	**Model 1**	**Model 2**	**Model 3**
6–7	2.10 (0.72–1.67, 0.64)	1.10 (0.72–1.68, 0.65)	1.09 (0.71–1.67, 0.68)
5–6	2.08 (1.34–3.25, <0.01)	2.11 (1.35–3.30, <0.01)	1.95 (1.24–3.07, <0.01)
<5	3.63 (2.09–6.28, <0.01)	3.54 (2.02–6.21, <0.01)	3.41 (1.94–6.04, <0.01)
*P* for trend	*P* < 0.01	*P* < 0.01	*P* < 0.01
**Male (*****n*** **= 1056)**			
6–7	1.27 (0.77–2.08, 0.33)	1.28 (0.78–2.12, 0.32)	1.30 (0.78–2.14, 0.30)
5–6	2.11 (1.24–3.59, <0.01)	2.13 (1.24–3.65, <0.01)	2.05 (1.19–3.52, <0.01)
<5	4.00 (2.06–7.75, <0.01)	4.04 (2.06–7.95, <0.01)	4.07 (2.06–8.01, <0.01)
*P* for trend	*P* < 0.01	*P* < 0.01	*P* < 0.01
**Female (*****n*** **= 390)**			
6–7	0.79 (0.35–1.76, 0.57)	0.77 (0.34–1.75, 0.54)	0.64 (0.27–1.51, 0.31)
5–6	2.04 (0.91–4.59, 0.08)	1.83 (0.79–4.20, 0.15)	1.33 (0.55–3.22, 0.51)
<5	2.93 (1.10–7.79, 0.03)	2.09 (0.70–6.27, 0.18)	1.52 (0.48–4.78, 0.47)
*P* for trend	*P* = 0.01	*P* = 0.10	*P* = 0.41
*P* for interaction^*^	*P* = 0.75	*P* = 0.76	*P* = 0.68

*Model 1: Adjusted for age and gender*.

*Model 2: Adjusted for Model 1 + marital status, education, monthly income, current smoker, current drinker, physical activity, body mass index, history of hypertension, hyperlipidemia, diabetes, heart disease, and migraine*.

*Model 3: Adjusted for Model 2 + NIHSS and ward type/specialty during hospitalization*.

* *P for interaction: analyses were stratified by gender*.

In men, the adjusted ORs in Model 3 were 1.30 (95% CI, 0.78–2.14), 2.05 (1.19–3.52), and 4.07 (2.06–8.01) for sleep durations of 6–7 h, 5–6 h, and <5 h, respectively. In women, the respective adjusted ORs were 0.64 (0.27–1.51), 1.33 (0.55–3.22), and 1.52 (0.48–4.78). In interaction analysis, the effect of sleep duration on PSA was not influenced by genders (*P* for interaction = 0.68) (Table 2).

Patients in other ward types/specialties, such as the intervention and medical departments, were less likely to develop PSA compared to those admitted in the stroke unit or ICU (OR, 0.52; 95% CI, 0.35–0.78, *P* < 0.01) (Supplementary Table 3).

### Impact of Sleep Duration on PSD at 3 Months

At 3 months after stroke, 254 patients (17.6%) had a PHQ-9 score of ≥5. For all participants, those with 5–6 and <5 h of sleep had a higher risk of PSD compared to those who slept for >7 h in Model 3 (OR, 1.47; 95% CI, 1.00–2.17; *P* = 0.04; and OR, 3.05; 95% CI, 1.85–5.02; *P* < 0.01, respectively). However, no difference was found between the groups with 6–7 and >7 h of sleep (OR, 0.92; 95% CI, 0.64–1.30; *P* = 0.64). A significant inverse correlation was also observed between prevalence of PSD and sleep duration (*P* for trend <0.01) (Table 3), which persisted after adjustment with propensity score matching (Supplementary Table 4).

**Table 3:** Impact of sleep duration on depression at 3 months after stroke.

**Sleep duration (hour)**	**Odds ratio (95% confidence interval**, ***P*****-value)**		
**Total (*n* = 1,446)**	**Model 1**	**Model 2**	**Model 3**
6–7	0.93 (0.65–1.31, 0.67)	0.92 (0.65–1.31, 0.67)	0.92 (0.64–1.30, 0.64)
5–6	1.65 (1.13–2.41, <0.01)	1.60 (1.10–2.35, 0.01)	1.47 (1.00–2.17, 0.04)
<5	3.28 (2.02–5.32, <0.01)	3.10 (1.89–5.07, <0.01)	3.05 (1.85–5.02, <0.01)
*P* for trend	*P* < 0.01	*P* < 0.01	*P* < 0.01
**Male (*****n*** **= 1,056)**			
6–7	1.08 (0.72–1.62, 0.68)	1.07 (0.71–1.61, 0.72)	1.08 (0.72–1.63, 0.68)
5–6	1.46 (0.92–2.30, 0.10)	1.41 (0.89–2.23, 0.14)	1.35 (0.85–2.15, 0.19)
<5	3.23 (1.80–5.81, <0.01)	3.09 (1.71–5.60, <0.01)	3.11 (1.71–5.66, <0.01)
*P* for trend	*P* < 0.01	*P* < 0.01	*P* < 0.01
**Female (*****n*** **= 390)**			
6–7	0.61 (0.30–1.22, 0.16)	0.70 (0.34–1.44, 0.33)	0.65 (0.30–1.40, 0.25)
5–6	2.17 (1.10–4.29, 0.02)	2.31 (1.13–4.69, 0.02)	1.68 (0.78–3.59, 0.18)
<5	3.35 (1.42–7.91, <0.01)	3.60 (1.39–9.30, <0.01)	2.96 (1.09–7.97, 0.03)
*P* for trend	*P* < 0.01	*P* < 0.01	*P* = 0.03
*P* for interaction^*^	*P* = 0.24	*P* = 0.25	*P* = 0.29

*Model 1: Adjusted for age and gender*.

*Model 2: Adjusted for Model 1 + marital status, education, monthly income, current smoker, current drinker, physical activity, body mass index, history of hypertension, hyperlipidemia, diabetes, heart disease, and migraine*.

*Model 3: Adjusted for Model 2 + NIHSS and ward type/specialty during hospitalization*.

* *P for interaction: analyses were stratified by gender*.

The adjusted ORs in Model 3 were 1.08 (95% CI, 0.72–1.63), 1.35 (0.85–2.15), and 3.11 (1.71–5.66) for sleep durations of 6–7 h, 5–6 h, <5 h for male patients, respectively, while the corresponding adjusted ORs for female patients were 0.65 (95% CI, 0.30–1.40), 1.68 (95% CI, 0.78–3.59), and 2.96 (95% CI, 1.09–7.97). Again, interaction analysis showed no effect of gender on the association between sleep duration and PSD (*P* for interaction = 0.29) (Table 3).

Patients with high NIHSS score (OR, 1.05; 95% CI, 1.01–1.10; *P* = 0.01) or admitted in the stroke unit (other wards vs. stroke unit; OR, 0.55; 95% CI, 0.39–0.78; *P* < 0.01) were at added risk for PSD (Supplementary Table 5).

## Discussion

In this study, the incidences of PSA and PSD in this study were 11.2 and 17.6% at 3 months after ischemic stroke, respectively. Patients with sleep duration of <6 h were more prone to PSA and PSD compared to those sleeping for >7 h, which did not differ between men and women. To the best of our knowledge, this analysis may represent the first multicentered prospective study that evaluates the impact of sleep duration on anxiety and depression after ischemic stroke. The large number of patients from 40 centers is representative of the demographic features of the Chinese population. Of note, the information was collected by face-to-face interview at baseline and 3 months after ischemic stroke, which guarantees the accuracy of data in this study.

The prevalence of PSA and PSD in our study was lower compared to other reports on the Chinese population, which were 15.1 and 21.1% at 3 months after ischemic stroke (20, 21). This inconsistency may be ascribed to the different test scales of anxiety and depressive symptoms, time frame, and study criteria. In this study, we excluded patients with history of stroke and sleep disorder who are at higher risks for PSA and PSD. This may be another reason for the lower prevalence of PSA and PSD in this series.

The literature has shown that self-reported short sleep duration was significantly correlated with PSA (22), sleep loss, and experimental sleep deprivation are linked to increased negative emotions (such as depression, madness, and nervousness) (23), and treatment of insomnia helps relieve the symptoms of anxiety (24). In line with previous studies, we also found that short sleep duration (<6 h) significantly increased the risk of PSA at 3 months compared with sleep duration of >7 h.

The underlying mechanisms for the association between short sleep duration and PSA are yet fully understood. Short sleep duration may affect emotional regulation by disturbing the functions of prefrontal cortex, anterior cingulate cortex, amygdala, and striatum. It can also disrupt the reward system in the brain, increase the cortisol level, unbalance the hypothalamus pituitary adrenal axis, and aggravate inflammatory reaction by raising the level of tumor necrosis factor-α (TNF-α), interleukin-6 (IL-6), and C-reactive protein (CRP). All these changes are secondary to sleep loss and will worsen the degree of anxiety (25). Another mechanism is that sleep deprivation reduced the availability of dopamine D2/D3 receptor in the striatum, since dopamine is a monoamine neurotransmitter playing an important role in the reward system and pleasure experience (26).

Currently available data are mixed on the association between sleep duration and depression. Previous studies have shown that in those aged 18–30 or >45 years, only short sleep duration (<6 h) posed a significantly high risk for depression, while long sleep duration (>9 h) did not (6, 27). Short sleep duration during weekdays was also found to increase the risk for depression in late adolescents (28). However, a U-shaped association between sleep duration and depression was found, in which both <5 and >9 h of sleep increased the risk of depression (29). In stroke patients, sleeping for <6 h were more likely to suffer depression than those sleeping for 7–8 h, while those with >8 h of sleep were not (30). This study also identified short sleep duration (<6 h) as a risk factor for PSD at 3 months. These conflicting results probably resulted from the difference in age and comorbidity among these studies. The mechanisms are complex. Possible mechanism may include chronic sleep restriction causing depression by disturbing the 5-hydroxytryptamine (5-HT) pathway and hypothalamic–pituitary–adrenal (HPA) axis (31) and sleep disruption leading to poor functional recovery, which increases the risk of depression (32).

It remains unclear if a gender difference exists in the association between sleep duration and anxiety or depression. In patients undergoing percutaneous transluminal coronary angioplasty, female patients had more difficulty initiating sleep and worse health-related quality of life, including anxiety and depression (33). Another study in elderly participants found that short sleep duration was associated with depression only in male patients, but no gender difference existed in the association of sleep duration and anxiety (34). Up to date, no prospective study is available on gender difference in the association between sleep duration and PSA or PSD, while ischemic stroke showed a higher incidence in male patients (35). Although this study found that a short duration of sleep was associated with increased risk for PSA in men, interaction analysis, on the contrary, showed that the impact of gender on the association between sleep duration and PSA was not significant. These inconsistent results may be ascribed to the relatively low proportion of female patients (27%) in this study, which also limits the power of statistical analysis. Some other confounding factors may exist that offset the risk of PSA in female patients and mask the interaction of gender with the impact of sleep duration on PSA, such as sleep biology and social pressure (36). Further studies are warranted in a large patient population of AIS with higher percentage of female patients to elucidate the interaction of gender with the impact of sleep duration.

### Limitations

This study has several limitations. First, the duration of sleep was calculated by the average sleep time during the past month prior to onset of stroke, which may not completely represent the sleep duration in acute phase of stroke, although sleep duration does not change easily. Second, the sleep duration was self-reported by patients rather than measured by polysomnography, which was neither feasible nor available in this multicenter study. Self-reported sleep duration is inaccurate with many inherent biases, especially to a study on anxiety and depression. Lastly, the duration of sleep in PSQI was taken as a categorical variable rather than a continuous one, which precludes the possibility of assessing the association between sleep duration and post-stroke anxiety or depression in a more accurate and quantitative manner.

## Conclusions

This study shows that short sleep duration (<6 h) was a predictor for anxiety and depression at 3 months after ischemic stroke. These results highlight the importance of the sleep duration in patients with ischemic stroke and argue for the need of monitoring patient sleep and ensuring adequate sleep duration (>6 h) to avoid or minimize the occurrence of post-stroke anxiety and depression.

## Data Availability Statement

The data analyzed in this study is subject to the following licenses/restrictions: The raw database is not public. Requests to access these datasets should be directed to http://paper.ncrcnd.ttctrc.com/default/project-detail?id=253.

## Ethics Statement

The studies involving human participants were reviewed and approved by medical ethics committees of Beijing Tiantan Hospital, Capital Medical University. The patients/participants provided their written informed consent to participate in this study.

## Author Contributions

Y-JW, C-XW, NZ, and X-LL designed the study. SW, X-LZ, and A-XW analyzed the data. FL and YY drafted the manuscript. H-JF, YQ, and W-GM revised the manuscript. All authors contributed to the article and approved the submitted version.

## Conflict of Interest

The authors declare that the research was conducted in the absence of any commercial or financial relationships that could be construed as a potential conflict of interest.

## References

[1] Wolfe CD Crichton SL Heuschmann PU McKevitt CJ Toschke AM Grieve AP. Estimates of outcomes up to ten years after stroke: analysis from the prospective South London Stroke Register. PLoS Med. (2011) 8:e1001033. 10.1371/journal.pmed.1001033 21610863 PMC3096613

[2] Bartoli F Lillia N Lax A Crocamo C Mantero V Carrà G. Depression after stroke and risk of mortality: a systematic review and meta-analysis. Stroke Res Treat. (2013) 2013:862978. 10.1155/2013/862978 23533964 PMC3606772

[3] House A Knapp P Bamford J Vail A. Mortality at 12 and 24 months after stroke may be associated with depressive symptoms at 1 month. Stroke. (2001) 32:696–701. 10.1161/01.STR.32.3.696 11239189

[4] Unsworth DJ Mathias JL Dorstyn DS. Preliminary screening recommendations for patients at risk of depression and/or anxiety more than 1 year poststroke. J Stroke Cerebrovasc Dis. (2019) 28:1519–28. 10.1016/j.jstrokecerebrovasdis.2019.03.014 30928216

[5] Watson NF Badr MS Belenky G Bliwise DL Buxton OM Buysse D. Recommended amount of sleep for a healthy adult: a joint consensus statement of the American Academy of Sleep Medicine and Sleep Research Society. Sleep. (2015) 38:843–4. 10.5665/sleep.4716 26039963 PMC4434546

[6] Sun Y Shi L Bao Y Sun Y Shi J Lu L. The bidirectional relationship between sleep duration and depression in community-dwelling middle-aged and elderly individuals: evidence from a longitudinal study. Sleep Med. (2018) 52:221–9. 10.1016/j.sleep.2018.03.011 29861378

[7] Ayerbe L Ayis SA Crichton S Wolfe CD Rudd AG. Natural history, predictors and associated outcomes of anxiety up to 10 years after stroke: the South London Stroke Register. Age Ageing. (2014) 43:542–7. 10.1093/ageing/aft208 24375225

[8] MacHale SM O'Rourke SJ Wardlaw JM Dennis MS. Depression and its relation to lesion location after stroke. J Neurol Neurosurg Psychiatry. (1998) 64:371–4. 10.1136/jnnp.64.3.371 9527152 PMC2170020

[9] Davis JC Falck RS Best JR Chan P Doherty S Liu-Ambrose T. Examining the inter-relations of depression, physical function, and cognition with subjective sleep parameters among stroke survivors: a cross-sectional analysis. J Stroke Cerebrovasc Dis. (2019) 28:2115–23. 10.1016/j.jstrokecerebrovasdis.2019.04.010 31129108

[10] Rickards H. Depression in neurological disorders: parkinson's disease, multiple sclerosis, and stroke. J Neurol Neurosurg Psychiatry. (2005) 76:i48–52. 10.1136/jnnp.2004.060426 15718222 PMC1765679

[11] Wang Y Jing J Xia M Pan Y Wang Y Zhao X. The Third China National Stroke Registry (CNSR-III) for patients with acute ischaemic stroke or transient ischaemic attack: design, rationale and baseline patient characteristics. Stroke Vasc Neurol. (2019) 4:158–64. 10.1136/svn-2019-000242 31709123 PMC6812638

[12] Liao XL Zuo LJ Zhang N Yang Y Pan YS Xiang XL. The occurrence and longitudinal changes of cognitive impairment after acute ischemic stroke. Neuropsychiatr Dis Treat. (2020) 16:807–14. 10.2147/NDT.S234544 32273707 PMC7114937

[13] Buysse DJ Reynolds CF Monk TH Berman SR Kupfer DJ. The Pittsburgh sleep quality index: a new instrument for psychiatric practice and research. Psychiatry Res. (1989) 28:193–213. 10.1016/0165-1781(89)90047-4 2748771

[14] Spitzer RL Kroenke K Williams JBW Löwe B. A brief measure for assessing generalized anxiety disorder: the GAD-7. Arch Intern Med. (2006) 166:1092–7. 10.1001/archinte.166.10.1092 16717171

[15] Kroenke K Spitzer RL Williams JB. The PHQ-9: validity of a brief depression severity measure. J Gen Intern Med. (2001) 16:6060–13. 10.1046/j.1525-1497.2001.016009606.x 11556941 PMC1495268

[16] Wang H Gong L Xia X Dong Q Jin A Gu Y. Red blood cell indices in relation to post-stroke psychiatric disorders: a longitudinal study in a follow-up stroke clinic. Curr Neurovasc Res. (2020) 17:218–23. 10.2174/1567202617666200423090958 32324513

[17] Ayasrah SM Ahmad MM Basheti IA. Post-stroke depression in Jordan: prevalence correlates and predictors. J Stroke Cerebrovasc Dis. (2018) 27:1134–42. 10.1016/j.jstrokecerebrovasdis.2017.11.027 29289425

[18] Carnes-Vendrell A Deus J Molina-Seguin J Pifarré J Purroy F. Depression and apathy after transient ischemic attack or minor stroke: prevalence, evolution and predictors. Sci Rep. (2019) 9:16248. 10.1038/s41598-019-52721-5 31700058 PMC6838079

[19] Sever S Doherty P Golder S Harrison AS. Is improvement in depression in patients attending cardiac rehabilitation with new-onset depressive symptoms determined by patient characteristics? Open Heart. (2020) 7:e001264. 10.1136/openhrt-2020-001264 32847994 PMC7451288

[20] Li W Xiao WM Chen YK Qu JF Liu YL Fang XW. Anxiety in patients with acute ischemic stroke: risk factors and effects on functional status. Front Psychiatry. (2019) 10:257. 10.3389/fpsyt.2019.00257 31057444 PMC6478797

[21] Liang Y Chan YL Deng M Chen YK Mok V Wang F. Enlarged perivascular spaces in the centrum semiovale are associated with poststroke depression: a 3-month prospective study. J Affect Disord. (2018) 228:166–72. 10.1016/j.jad.2017.11.080 29253682

[22] Almhdawi KA Alazrai A Kanaan S Shyyab AA Oteir AO Mansour ZM. Post-stroke depression, anxiety, and stress symptoms and their associated factors: a cross-sectional study. Neuropsychol Rehabil. (2020) 18:1–14. 10.1080/09602011.2020.1760893 32419606

[23] Palmer CA Alfano CA. Sleep and emotion regulation: an organizing, integrative review. Sleep Med Rev. (2017) 31:6–16. 10.1016/j.smrv.2015.12.006 26899742

[24] Gosling JA Batterham P Ritterband L Glozier N Thorndike F Griffiths KM. Online insomnia treatment and the reduction of anxiety symptoms as a secondary outcome in a randomised controlled trial: the role of cognitive-behavioural factors. Aust N Z J Psychiatry. (2018) 52:1183–93. 10.1177/0004867418772338 29717621

[25] Blake MJ Trinder JA Allen NB. Mechanisms underlying the association between insomnia, anxiety, and depression in adolescence: implications for behavioral sleep interventions. Clin Psychol Rev. (2018) 63:25–40. 10.1016/j.cpr.2018.05.006 29879564

[26] Volkow ND Tomasi D Wang GJ Telang F Fowler JS Logan J. Evidence that sleep deprivation downregulates dopamine D2R in ventral striatum in the human brain. J Neurosci. (2012) 32:6711–7. 10.1523/JNEUROSCI.0045-12.2012 22573693 PMC3433285

[27] Montagni I Qchiqach S Pereira E Tully PJ Tzourio C. Sex-specific associations between sleep and mental health in university students: a large cross-sectional study. J Am Coll Health. (2020) 68:278–85. 10.1080/07448481.2018.1546183 30615574

[28] Koo DL Yang KI Kim JH Kim D Sunwoo JS Hwangbo Y. Association between morningness-eveningness, sleep duration, weekend catch-up sleep and depression among Korean high-school students. J Sleep Res. (2020) 30:e13063. 10.1111/jsr.13063 32391631

[29] Guo L Wang T Wang W Fan B Xie B Zhang H. Association between habitual weekday sleep duration and depressive symptoms among Chinese adolescents:The role of mode of birth delivery. J Affect Disord. (2020) 265:583–9. 10.1016/j.jad.2019.11.095 31759667

[30] Dong L Brown DL Chervin RD Case E Morgenstern LB Lisabeth LD. Pre-stroke sleep duration and post-stroke depression. Sleep Med. (2020) 77:325–9. 10.1016/j.sleep.2020.04.025 32828696 PMC7667889

[31] Novati A Roman V Cetin T Hagewoud R den Boer JA Luiten PG. Chronically restricted sleep leads to depression-like changes in neurotransmitter receptor sensitivity and neuroendocrine stress reactivity in rats. Sleep. (2008) 31:1579–85. 10.1093/sleep/31.11.1579 19014078 PMC2579986

[32] Duss SB Seiler A Schmidt MH Pace M Adamantidis A Müri RM. The role of sleep in recovery following ischemic stroke: a review of human and animal data. Neurobiol Sleep Circadian Rhythms. (2017) 2:94–105. 10.1016/j.nbscr.2016.11.003 31236498 PMC6575180

[33] Edéll-Gustafsson UM Hetta JE. Fragmented sleep and tiredness in males and females one year after percutaneous transluminal coronary angioplasty (PTCA). J Adv Nurs. (2001) 34:203–11. 10.1046/j.1365-2648.2001.01746.x 11430282

[34] Brostrom A Wahlin A Alehagen U Ulander M Johansson P. Sex-specific associations between self-reported sleep duration, depression, anxiety, fatigue and daytime sleepiness in an older community-dwelling population. Scand J Caring Sci. (2018) 32:290–8. 10.1111/scs.12461 28574585

[35] Koton S Schneider AL Rosamond WD Shahar E Sang Y Gottesman RF. Stroke incidence and mortality trends in US communities, 1987 to 2011. JAMA. (2014) 312:259–68. 10.1001/jama.2014.7692 25027141

[36] Pase MP. The association between sleep duration and stroke differs by race and sex. Neurology. (2018) 91:e1728–31. 10.1212/WNL.0000000000006420 30373931

