# Supplementary material for: Impact of Sleep Duration on Depression and Anxiety After Acute Ischemic Stroke
Source: Front Neurol. 2021 Mar 26;12:630638. doi: 10.3389/fneur.2021.630638 (PMC8032928; doi:10.3389/fneur.2021.630638)
Supplement: Supplementary file 1 [file Table_1.docx]

**Supplemental table 1. Inter-group analysis**

| Variable/sleep duration (hours) | P value |
| --- | --- |
| Age |  |
| >7 and 6-7 | 0.68 |
| >7 and 5-6 | <0.01 |
| >7 and <5 | 0.27 |
| 6-7 and 5-6 | <0.01 |
| 6-7 and <5 | 0.20 |
| 5-6 and <5 | 0.53 |
| Education |  |
| >7 and 6-7 | <0.01 |
| >7 and 5-6 | 0.15 |
| >7 and <5 | 0.04 |
| 6-7 and 5-6 | 0.53 |
| 6-7 and <5 | 0.58 |
| 5-6 and <5 | 0.35 |
| Hypertension history |  |
| >7 and 6-7 | 0.58 |
| >7 and 5-6 | 0.34 |
| >7 and <5 | <0.01 |
| 6-7 and 5-6 | 0.64 |
| 6-7 and <5 | 0.01 |
| 5-6 and <5 | 0.03 |
| Migraine history |  |
| >7 and 6-7 | 0.02 |
| >7 and 5-6 | 0.63 |
| >7 and <5 | <0.01 |
| 6-7 and 5-6 | 0.21 |
| 6-7 and <5 | 0.05 |
| 5-6 and <5 | <0.01 |
| Stroke family history |  |
| >7 and 6-7 | 0.16 |
| >7 and 5-6 | 0.15 |
| >7 and <5 | <0.01 |
| 6-7 and 5-6 | 0.02 |
| 6-7 and <5 | 0.01 |
| 5-6 and <5 | <0.01 |
| Motor disorder |  |
| >7 and 6-7 | 0.47 |
| >7 and 5-6 | <0.01 |
| >7 and <5 | 0.17 |
| 6-7 and 5-6 | 0.02 |
| 6-7 and <5 | 0.34 |
| 5-6 and <5 | 0.54 |
| Ward type/specialty during hospitalization |  |
| >7 and 6-7 | 0.91 |
| >7 and 5-6 | <0.01 |
| >7 and <5 | 0.21 |
| 6-7 and 5-6 | <0.01 |
| 6-7 and <5 | 0.35 |
| 5-6 and <5 | 0.50 |
| Depression at baseline |  |
| >7 and 6-7 | 0.81 |
| >7 and 5-6 | <0.01 |
| >7 and <5 | <0.01 |
| 6-7 and 5-6 | <0.01 |
| 6-7 and <5 | <0.01 |
| 5-6 and <5 | <0.01 |
| Anxiety at baseline |  |
| >7 and 6-7 | 0.85 |
| >7 and 5-6 | <0.01 |
| >7 and <5 | <0.01 |
| 6-7 and 5-6 | 0.01 |
| 6-7 and <5 | <0.01 |
| 5-6 and <5 | <0.01 |
