# Supplementary material for: Impact of Sleep Duration on Depression and Anxiety After Acute Ischemic Stroke
Source: Front Neurol. 2021 Mar 26;12:630638. doi: 10.3389/fneur.2021.630638 (PMC8032928; doi:10.3389/fneur.2021.630638)
Supplement: Supplementary file 2 [file Table_2.docx]

**Supplemental table 2. Adjusted multivariable analysis of post-stroke anxiety**

| Variable | Odds ratio (95% confidence interval) | P value |
| --- | --- | --- |
| 6-7 hours sleep duration | 1.06 (0.82-1.37) | 0.60 |
| 5-6 hours sleep duration | 1.94 (1.53-2.45) | <0.01 |
| <5 hours sleep duration | 3.07 (2.45-3.84) | <0.01 |
| Age, year | 0.99 (0.98-0.99) | 0.01 |
| Female gender | 0.75 (0.61-0.92) | <0.01 |
| Married | 1.54 (1.01-2.34) | 0.04 |
| Education ≥ high school | 1.23 (1.03-1.47) | 0.02 |
| High monthly income | 0.51 (0.43-0.62) | <0.01 |
| Current smoker | 0.62 (0.50-0.75) | <0.01 |
| Current drinker | 0.82 (0.65-1.03) | 0.09 |
| Physical activity | 1.13 (0.95-1.36) | 0.15 |
| Body mass index, kg/m^2^ | 0.97 (0.94-1.00) | 0.05 |
| Hypertension | 1.38 (1.15-1.65) | <0.01 |
| Hyperlipidemia | 1.28 (0.98-1.67) | 0.06 |
| Diabetes | 0.83 (0.68-1.02) | 0.08 |
| Heart disease | 1.10 (0.87-1.40) | 0.39 |
| Migraine | 4.69 (3.20-6.89) | <0.01 |
| NIHSS at baseline | 1.02 (0.99-1.04) | 0.09 |
| Other wards vs stroke unit | 0.49 (0.40-0.59) | <0.01 |
| ICU vs stroke unit | 0.99 (0.56-1.75) | 0.99 |

NIHSS, National Institutes of Health Stroke Scale; ICU, Intensive Care Unit; Other wards, wards/specialties exclusive of the stroke unit and ICU
