# Supplementary material for: Impact of Sleep Duration on Depression and Anxiety After Acute Ischemic Stroke
Source: Front Neurol. 2021 Mar 26;12:630638. doi: 10.3389/fneur.2021.630638 (PMC8032928; doi:10.3389/fneur.2021.630638)
Supplement: Supplementary file 3 [file Table_3.docx]

**Supplemental table 3. Logistic regression for post-stroke anxiety at 3 months**

| Variable | Odds ratio (95% confidence interval) | P value |
| --- | --- | --- |
| Age, year | 0.98 (0.96-1.00) | 0.07 |
| Female gender | 1.02 (0.67-1.55) | 0.91 |
| Married | 1.43 (0.60-3.44) | 0.41 |
| Education ≥ high school | 0.96 (0.65-1.41) | 0.85 |
| High monthly income | 0.68 (0.47-1.00) | 0.05 |
| Current smoker | 0.74 (0.48-1.12) | 0.16 |
| Current drinker | 0.94 (0.58-1.51) | 0.81 |
| Physical activity | 0.99 (0.69-1.43) | 0.97 |
| Body mass index, kg/m^2^ | 0.97 (0.91-1.02) | 0.27 |
| Hypertension | 1.07 (0.75-1.53) | 0.68 |
| Hyperlipidemia | 1.13 (0.62-2.04) | 0.67 |
| Diabetes | 1.01 (0.66-1.53) | 0.94 |
| Heart disease | 0.95 (0.55-1.62) | 0.85 |
| Migraine | 2.11 (0.78-5.69) | 0.13 |
| NIHSS at baseline | 1.01 (0.96-1.06) | 0.64 |
| Other wards vs stroke unit | 0.52 (0.35-0.78) | <0.01 |
| ICU vs stroke unit | 1.25 (0.42-3.71) | 0.68 |

NIHSS, National Institutes of Health Stroke Scale; ICU, Intensive Care Unit; Other wards, wards/specialties exclusive of the stroke unit and ICU
