# Supplementary material for: Impact of Sleep Duration on Depression and Anxiety After Acute Ischemic Stroke
Source: Front Neurol. 2021 Mar 26;12:630638. doi: 10.3389/fneur.2021.630638 (PMC8032928; doi:10.3389/fneur.2021.630638)
Supplement: Supplementary file 4 [file Table_4.docx]

**Supplemental table 4. Adjusted multivariable analysis of post-stroke depression**

| Variable | Odds ratio (95% confidence interval) | P value |
| --- | --- | --- |
| 6-7 hours sleep duration | 0.91 (0.74-1.12) | 0.40 |
| 5-6 hours sleep duration | 1.45 (1.19-1.77) | <0.01 |
| <5 hours sleep duration | 2.95 (2.45-3.56) | <0.01 |
| Age, year | 1.00 (1.00-1.01) | 0.01 |
| Female gender | 1.00 (0.84-1.20) | 0.92 |
| Married | 1.86 (1.30-2.66) | <0.01 |
| Education ≥ high school | 1.09 (0.93-1.27) | 0.26 |
| High monthly income | 0.81 (0.70-0.95) | <0.01 |
| Current smoker | 0.92 (0.78-1.09) | 0.38 |
| Current drinker | 0.92 (0.76-1.11) | 0.39 |
| Physical activity | 1.28 (1.10-1.50) | <0.01 |
| Body mass index, kg/m^2^ | 0.96 (0.93-0.98) | <0.01 |
| Hypertension | 1.18 (1.02-1.38) | 0.02 |
| Hyperlipidemia | 1.75 (1.40-2.19) | <0.01 |
| Diabetes | 1.00 (0.85-1.18) | 0.94 |
| Heart disease | 1.59 (1.31-1.93) | <0.01 |
| Migraine | 1.90 (1.28-2.82) | <0.01 |
| NIHSS at baseline | 1.06 (1.04-1.08) | <0.01 |
| Other wards vs stroke unit | 0.46 (0.39-0.54) | <0.01 |
| ICU vs stroke unit | 1.51 (0.95-2.40) | 0.07 |

NIHSS, National Institutes of Health Stroke Scale; ICU, Intensive Care Unit; Other wards, wards/specialties exclusive of the stroke unit and ICU
