# Supplementary material for: Impact of Sleep Duration on Depression and Anxiety After Acute Ischemic Stroke
Source: Front Neurol. 2021 Mar 26;12:630638. doi: 10.3389/fneur.2021.630638 (PMC8032928; doi:10.3389/fneur.2021.630638)
Supplement: Supplementary file 5 [file Table_5.docx]

**Supplemental table 5. Logistic regression for post-stroke depression at 3 months**

| Variable | Odds ratio (95% confidence interval) | P value |
| --- | --- | --- |
| Age, year | 1.01 (0.99-1.02) | 0.10 |
| Female gender | 1.11 (0.77-1.58) | 0.56 |
| Married | 1.77 (0.85-3.70) | 0.12 |
| Education ≥ high school | 1.02 (0.74-1.40) | 0.86 |
| High monthly income | 0.98 (0.72-1.33) | 0.92 |
| Current smoker | 1.03 (0.72-1.45) | 0.86 |
| Current drinker | 1.07 (0.73-1.57) | 0.69 |
| Physical activity | 1.03 (0.76-1.40) | 0.81 |
| Body mass index, kg/m^2^ | 0.96 (0.91-1.00) | 0.08 |
| Hypertension | 1.25 (0.92-1.69) | 0.14 |
| Hyperlipidemia | 1.39 (0.86-2.24) | 0.17 |
| Diabetes | 1.02 (0.72-1.44) | 0.88 |
| Heart disease | 1.21 (0.80-1.83) | 0.35 |
| Migraine | 1.35 (0.50-3.62) | 0.54 |
| NIHSS at baseline | 1.05 (1.01-1.10) | 0.01 |
| Other wards vs stroke unit | 0.55 (0.39-0.78) | <0.01 |
| ICU vs stroke unit | 1.50 (0.59-3.78) | 0.38 |

NIHSS, National Institutes of Health Stroke Scale; ICU, Intensive Care Unit; Other wards, wards/specialties exclusive of the stroke unit and ICU
